# Baseline peripheral blood neutrophil-to-lymphocyte ratio could predict survival in patients with adult polymyositis and dermatomyositis: A retrospective observational study

**DOI:** 10.1371/journal.pone.0190411

**Published:** 2018-01-02

**Authors:** You-Jung Ha, Jaehyung Hur, Dong Jin Go, Eun Ha Kang, Jin Kyun Park, Eun Young Lee, Kichul Shin, Eun Bong Lee, Yeong Wook Song, Yun Jong Lee

**Affiliations:** 1 Department of Internal Medicine, Seoul National University Bundang Hospital, Seongnam, Republic of Korea; 2 Department of Internal Medicine, Seoul National University Hospital, Seoul, Republic of Korea; 3 Department of Internal Medicine, Seoul National University College of Medicine, Seoul, Republic of Korea; 4 Department of Internal Medicine, Seoul Metropolitan Government–Seoul National University Boramae Medical Center, Seoul, Republic of Korea; 5 WCU Department of Molecular Medicine and Biopharmaceutical Sciences, Medical Research Institute, Seoul National University College of Medicine, Seoul, Republic of Korea; Keio University, JAPAN

## Abstract

Recent studies have suggested that neutrophil-to-lymphocyte ratio (NLR) and C-reactive protein-to-albumin ratio (CAR) are emerging markers of disease activity and prognosis in patients with chronic inflammatory diseases, cardiovascular diseases, or malignancies. Therefore, we investigated the clinical significance and prognostic value of the NLR and CAR in adult patients with polymyositis and dermatomyositis. The medical records of 197 patients with newly diagnosed polymyositis/dermatomyositis between August 2003 and November 2016 were retrospectively reviewed. Survival and causes of death were recorded during an average 33-month observational period. Clinical and laboratory findings were compared between survivors and non-survivors. Using receiver operating characteristic curves, the NLR and CAR cut-off values for predicting survival were calculated. Univariate and multivariate analyses using Cox proportional hazard models were performed to identify factors associated with survival. Twenty-six patients (13.2%) died during the study period, and the 5-year survival-rate was estimated to be 82%. The non-survivor group exhibited older age and a higher prevalence of interstitial lung disease (ILD), acute interstitial pneumonia, and acute exacerbation of ILD compared to that in the survivor group. NLR and CAR values were significantly higher in the non-survivors and in patients with polymyositis/dermatomyositis-associated ILD, and the death rates increased across NLR and CAR quartiles. Furthermore, when stratified according to the NLR or CAR optimal cut-off values, patients with a high NLR (>4.775) or high CAR (>0.0735) had a significantly lower survival rate than patients with low NLR or CAR, respectively. In addition, old age (>50 years), the presence of acute interstitial pneumonia, hypoproteinemia (serum protein <5.5 g/dL), and high NLR (but not high CAR) were independent predictors for mortality. The results indicate that a high NLR is independently associated with worse overall survival. Thus, the baseline NLR level may be a simple, cost-effective prognostic marker in patients with polymyositis/dermatomyositis.

## Introduction

Polymyositis (PM) and dermatomyositis (DM) are two classic forms of idiopathic inflammatory myopathy (IIM). They are characterized by symmetric proximal muscle weakness, myopathic electromyographic findings, elevated serum muscle enzymes, or mononuclear cell infiltrates with muscle fiber necrosis. DM is distinguished from PM by typical cutaneous manifestations and clinically amyopathic dermatomyositis (CADM) is a unique subset of DM without myositis. Apart from the skin involvement, IIM also can involve other organ systems, including the lung, heart, and joints [1]. Additionally, increased risk for malignancies in patients with PM/DM has been widely described, with the strongest association occurring in patient with DM.

In earlier studies, the prognosis of PM/DM was poor, with a 5-year survival rate of less than 50%; however, recent studies have shown improved survival [2–5]. Nonetheless, the overall mortality rate in patients with PM/DM remains two to three-fold higher compare to that for the general population [5]. Old age, delay in diagnosis or treatment, cancer-associated myositis, and the presence of several extra-muscular organ involvements including lung have been reported as clinical poor prognostic factors [1,5,6]. In particular, patients with PM/DM who experience an acute deterioration in interstitial lung disease (ILD) are more likely to die [7], and the prognosis of PM/DM associated acute interstitial pneumonia (AIP) is extremely unfavorable [8]. Furthermore, several studies have suggested that the serum levels of IL-6 and certain myositis-specific autoantibodies (e.g., anti-melanoma differentiation-associated gene (MDA) 5, anti-nuclear matrix protein (NXP)-2, and anti-transcriptional intermediary factor (TIF) 1-γ), could be useful biomarkers for predicting a poor prognosis [9]. However, these myositis-specific antibodies are not easily measurable in the clinical practice, and a valuable and simple prognostic biomarker has not yet been developed for patients with IIM.

Recently, the neutrophil-to-lymphocyte ratio (NLR), platelet-lymphocyte ratio (PLR), and C-reactive protein-to-albumin ratio (CAR) have been suggested as useful and cost-effective prognostic biomarkers in various diseases, including malignant and cardiovascular diseases [10–12]. In addition, NLR, PLR, or CAR was recently reported to serve useful inflammatory markers reflecting disease activity or inflammatory burden in patients with systemic rheumatic diseases including systemic lupus erythematosus, rheumatoid arthritis, and PM/DM [13–18]. However, their prognostic significance in PM/DM has not yet been evaluated. Therefore, in the present study, we investigated the clinical implications of the NLR, PLR, and CAR values and determined whether these measures can serve as independent prognostic biomarkers in patients with DM/PM.

## Materials and methods

### Methods

We retrospectively reviewed medical records from 225 patients who were newly diagnosed with PM/DM between August 2003 and November 2016 at the Seoul National University Bundang Hospital and Seoul National University Hospital, South Korea. The diagnosis of classical PM/DM was made according to the criteria of Bohan and Peter [19,20]. CADM was diagnosed based on the criteria proposed by Gerami *et al* [21]. Twenty-eight patients were excluded because of missing baseline data regarding the differential leukocyte count or C-reactive protein (CRP) level (n = 27), or the presence of the human immunodeficiency virus infection (n = 1). This study was approved by the Institutional Review Board (IRB) of Seoul National University Bundang Hospital and Seoul National University Hospital (IRB No. B-1609/362-105). Informed consent was waived, as the study involved a retrospective chart review with minimal risk to the patients. All data were anonymized prior to analysis.

### Data collection

Collected data included demographic variables, muscular symptoms, extra-muscular organ involvement, and the use of therapeutic medications, including glucocorticoids, cyclosporine, and cyclophosphamide. Baseline laboratory variables were collected, including the white blood cell count (WBC), absolute neutrophil count (ANC), absolute lymphocyte count (ALC), hemoglobin level, platelet count, erythrocyte sedimentation rate (ESR), CRP level, creatinine level, creatine phosphokinase (CPK) level, lactate dehydrogenase (LDH) level, anti-nuclear antibody, and anti-Jo-1 antibody.

The presence of ILD was confirmed by typical high-resolution computed tomography (HRCT) findings, which include irregular linear, reticular, or ground-glass opacities, consolidations, traction bronchiectasis, or honeycombing [22,23]. We also obtained the available pulmonary function test results, including the forced vital capacity (FVC) and diffusing capacity of the lung for carbon monoxide (DLco). The acute exacerbation of ILD (AE-ILD) was defined by worsening of dyspnea within 1 month, new bilateral opacities on HRCT, and the exclusion of other causes for the worsening symptoms in patients with ILD [24]. The survival status and causes of death during the follow-up period were ascertained from the hospital records.

### Definition of inflammation–based scores

Two hematological inflammatory indices, NLR and PLR, were calculated using hematological parameters (absolute count of neutrophil, lymphocyte, and platelet) measured by an automated analyzers: NLR = ANC/ALC and PLR = absolute platelet count/ALC. The CAR was calculated as: CAR = serum CRP level (mg/dL)/serum albumin level (g/dL).

### Statistical analysis

Data are presented as means ± standard deviation or as numbers (percentages). The NLR, PLR, and CAR were log transformed, as their distributions were non-normal. Group differences were evaluated using the Chi-square test or Fisher’s exact test for categorical data and *t*-test for continuous variables. Optimal cut-off values for predicting survival were extrapolated using the receiver operator characteristic (ROC) curves and the area under the ROC curve (AUC) was calculated. Kaplan-Meier analyses and log-rank test were used to evaluate group differences in the survival curves. For the multivariate analysis of overall survival, a Cox proportional hazards model was used with selective variables (those with P <0.1 in the univariate analyses, and known risk factors. Statistical analyses were performed using IBM SPSS software, version 23.0 (IBM Co., New York, USA) and STATA 14.0 (StataCorp., Texas, USA). A two-tailed P value < 0.05 was considered statistically significant.

## Results

### Characteristics of survivors and non-survivors

The baseline clinical and laboratory characteristics are presented in Table 1. A total of 115 patients (58.4%) had classical DM, 44 (22.3%) had PM, and 38 (19.3%) were diagnosed with CADM. The mean age at diagnosis was 50.3 years and 128 patients (65.0%) were women. Twenty-three patients (11.7%) detected malignancy within 1 year before or after the diagnosis of IIM and 101 patients (51.3%) had PM/DM-associated ILD. During a mean follow-up period of 33.6 ± 33.9 months (median, 20.3 months; range, 0.2–146.0 months), 26 patients (13.2%) died, and the 5-year survival rate was estimated to be 82%. Of the non-survivors, 20 patients (76.9%) died within 1 year after the diagnosis of IIM. Twenty-one (20.8%) of the 101 patients with ILD eventually succumbed, and 16 (40.0%) of the 40 patients with AE-ILD died during follow-up. Additionally, the death rate among 13 patients with AIP was very high (12/13, 92.3%). Finally, the causes of death for 19 (73.1%) of 26 non-survivors was secondary to pulmonary morbidity. Detailed causes of death are summarized in S1 Table.

**Table 1:** Demographic and clinical features of the study population.

	Total patients (n = 197)	Survivors (n = 171)	Non-survivors (n = 26)	*P* value
Male:Female	69:128	60:111	9:17	0.96
Age at diagnosis (years)	50.3 ± 14.0	48.8 ± 13.9	59.7 ± 10.5	<0.001
Age > 50 years	113 (57.4)	90 (52.6)	23 (88.5)	<0.001
Follow up period (months)	33.6 ± 33.9	37.2 ± 34.5	9.5 ± 14.5	<0.001
IIM subtypes				0.48
PM	44 (22.3)	40 (23.4)	4 (15.4)	
Classic DM	115 (58.4)	97 (56.7)	18 (69.2)	
CADM	38 (14.2)	34 (19.9)	4 (15.4)	
Overlap syndrome	30 (15.2)	27 (15.8)	3 (11.5)	0.77
Extra-muscular manifestations				
Heliotrope rash	50/153 (32.7)	42/131 (32.1)	8/22 (36.4)	0.69
Gottron's papule	91/153 (59.5)	75/131 (57.3)	16/22 (72.7)	0.17
V neck sign	64/153 (41.8)	56/131 (42.7)	8/22 (36.4)	0.57
Mechanic’s hand	22/153 (14.4)	21/131 (16.0)	1/22 (4.5)	0.20
Fever	35 (17.8)	27 (15.8)	8 (30.8)	0.06
Raynaud’s phenomenon	25 (12.7)	24 (14.0)	1 (3.8)	0.21
Arthralgia/Arthritis	77 (39.1)	68 (39.8)	9 (34.6)	0.62
Myocardial involvement	2 (1.0)	1 (0.6)	1 (3.8)	0.25
ILD	101 (51.3)	81 (47.4)	20 (76.9)	0.005
AIP	13/101 (12.9)	1/81 (1.2)	12/20 (60.0)	<0.001
Acute exacerbation of ILD	40/101 (39.6)	24/81 (29.6)	16/20 (61.4)	<0.001
Malignancy history				
> 2 years before IIM diagnosis	5 (2.5)	3 (2.9)	2 (7.7)	0.38
Within ± 2 year from IIM diagnosis	28 (14.2)	23 (13.5)	5 (19.2)	0.43
Within ± 1 year from IIM diagnosis	23 (11.7)	18 (10.5)	5 (19.2)	0.20
WBC, mm^3^	7268 ± 4045	7364 ± 3750	9362 ± 5392	0.08
ANC, mm^3^	5528 ± 3790	5206 ± 3498	7647 ± 4900	0.02
Neutrophilia (ANC >7500)	42 (21.3)	32 (18.7)	10 (38.5)	0.02
ALC, mm^3^	1369 ± 813	1432 ± 835	960 ± 490	<0.001
Lymphopenia (ALC <1000)	69 (35.0)	52 (30.4)	17 (65.4)	<0.001
Hemoglobin, g/dL	12.1 ± 1.7	12.2 ± 1.7	11.6 ± 1.9	0.10
Platelet, ×10^3^/mm^3^	254 ± 102	257 ± 101	239 ± 113	0.41
NLR, log-transformed	1.364 ± 0.841	1.266 ± 0.822	2.002 ± 0.684	<0.001
ESR, mm/hr (n = 193)	40 ± 24	39 ± 25	44 ± 18	0.27
CRP, mg/dL	1.94 ± 3.46	1.65 ± 3.10	3.90 ± 4.93	0.03
Albumin, g/dL	3.36 ± 0.53	3.43 ± 0.58	2.91 ± 0.76	<0.001
CAR, log-transformed	-2.176 ± 2.277	-2.403 ± 2.245	-0.687 ± 1.934	<0.001
Protein, g/dL	6.64 ± 0.86	6.72 ± 0.82	6.03 ± 0.89	<0.001
Creatinine, mg/dL	0.71 ± 0.42	0.68 ± 0.19	0.92 ± 1.03	0.25
CPK, IU/L	2661 ± 4793	2820 ± 5042	1614 ± 2435	0.05
LDH, IU/L (n = 187)	585 ± 471	596 ± 496	512 ± 225	0.42
Anti-Jo-1 positivity	26/178 (14.6)	24/152 (15.8)	2/26 (7.7)	0.38
Pulmonary function				
FVC (n = 130)	2.60 ± 0.87	2.60 ± 0.88	2.64 ± 0.73	0.88
FVC, percent predicted	74.7 ± 18.2	74.5 ± 18.2	75.9 ± 19.0	0.79
DLco (n = 121)	13.4 ± 5.6	13.6 ± 5.7	11.9 ± 4.0	0.28
DLco percent predicted	68.3 ± 22.3	68.3 ± 22.8	67.7 ±18.6	0.91
Treatment				
High-dose glucocorticoid*	162 (82.2)	138 (80.7)	24 (92.3)	0.18
Cyclosporine A	65 (33.0)	54 (31.6)	11 (42.3)	0.28
Cyclophosphamide	18 (9.1)	13 (7.6)	5 (19.2)	0.06

Values are presented as n (%) or mean ± standard deviation.

* > 30mg as the prednisolone equivalent dose a day

Abbreviation: IIM, idiopathic inflammatory myopathy; PM, polymyositis; DM, dermatomyositis; CADM, clinically amyopathic dermatomyositis; ILD, interstitial lung disease; AIP, acute interstitial pneumonia; WBC, white blood cells; ANC, absolute neutrophil count; ALC, absolute lymphocyte count; NLR, neutrophil-to-lymphocyte ratio; ESR, erythrocyte sedimentation rate; CRP, C-reactive protein; CAR, CRP-to-albumin ratio; CPK, creatine phosphokinase; LDH, lactate dehydrogenase; FVC, forced vital capacity; DLco, diffusion capacity for carbon monoxide.

Non-survivors were significantly older compared to survivors (59.7 ± 10.5 versus 48.8 ± 13.9 years, *P* < 0.001), and were more likely to have ILD (76.9% versus 47.4%, *P* = 0.005), acute interstitial pneumonia (AIP, 46.2% versus 0.6%, *P* < 0.001), and AE-ILD (61.4% versus 29.6%, *P* < 0.001). However, the groups were comparable in the prevalence of malignancy and fever (Table 1).

**Fig 1:**
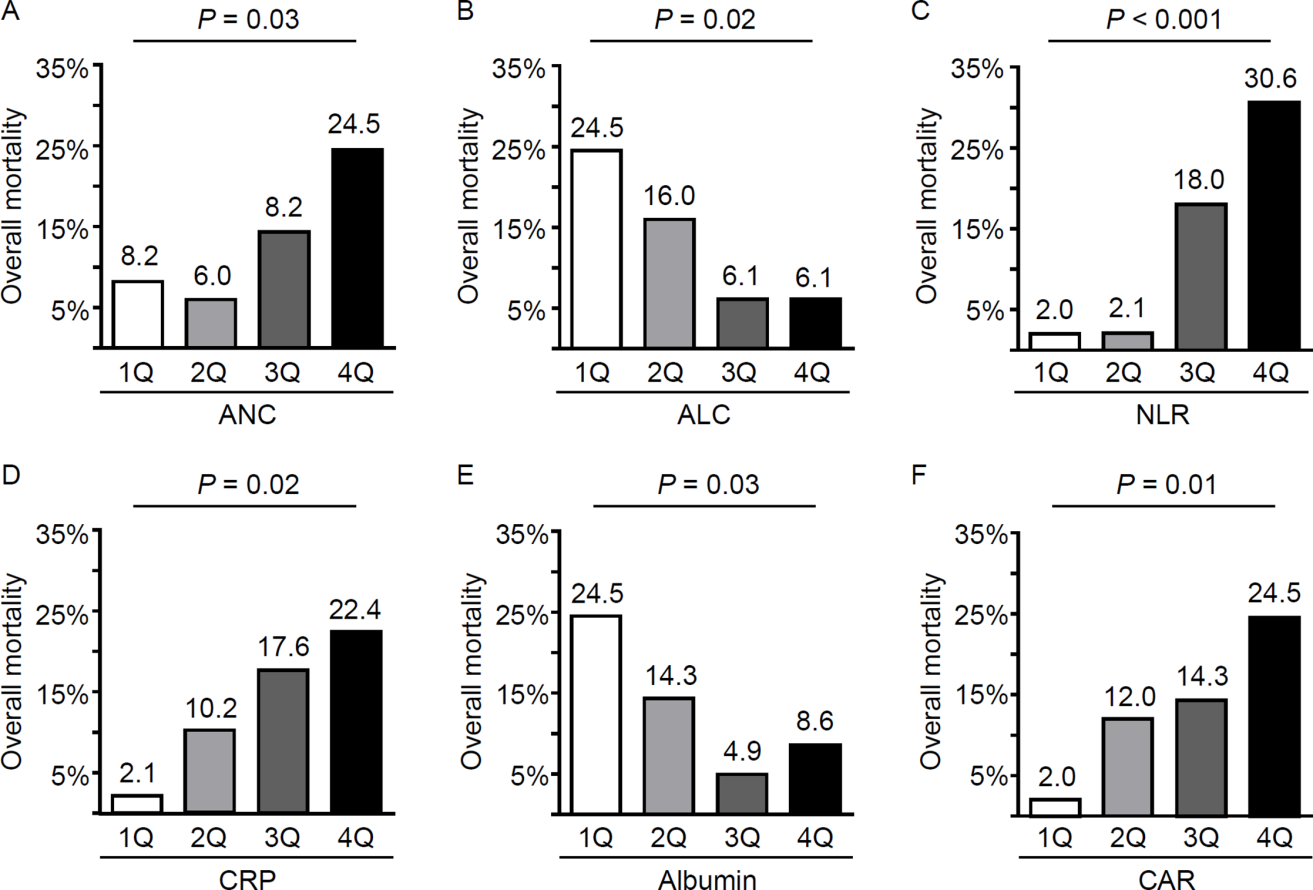
The distribution of non-survivors based on the quartiles of absolute neutrophil and lymphocyte counts, and serum CRP and albumin levels in patients with polymyositis/dermatomyositis. The prevalence of non-survivors increased across quartiles for absolute neutrophil count (ANC, A), and decreased across quartiles for absolute lymphocyte count (ALC, B). Consequently, the prevalence of non-survivors was the largest in the highest quartile of the neutrophil/lymphocyte ratio (NLR, C). Also, the prevalence of non-survivor increased with increasing quartiles of C-reactive protein (CRP, D), and with lower quartiles for albumin (E). Consequently, the distribution of non-survivors was significantly different among CRP/albumin ratio (CAR) quartiles (F). *P* values were calculated by chi-square test.

Additionally, non-survivors had significantly higher ANC (7647 ± 4900 versus 5206 ± 3498/mm^3^, *P* < 0.05) and lower ALC (960 ± 490 versus 1432 ± 835/mm^3^, *P* < 0.001) compared to those in survivors, even though the groups were comparable in WBC counts (Table 1). The baseline CRP levels were significantly higher in non-survivors compared to that in survivors (3.90 ± 4.93 versus 1.65 ± 3.10 mg/dL, *P* < 0.05), while the serum total protein (6.03 ± 0.89 versus 6.72 ± 0.82 g/dL, *P* < 0.001) and albumin levels (2.91 ± 0.76 versus 3.43 ± 0.58, *P* < 0.001) were significantly lower in non-survivors compared to those in survivors. Moreover, the distribution of the overall mortality rates significantly differed according to ANC, ALC, CRP, and albumin quartiles (Fig 1).

### Clinical implication of NLR and CAR in patients with IIM

Because the NLR and CAR have been previously reported as prognostic predictors in various diseases [10–12], and the distribution of non-survivors was significantly different across NLR and CAR quartiles in the present study (Fig 1), the clinical significance of these two indices was further evaluated.

**Fig 2:**
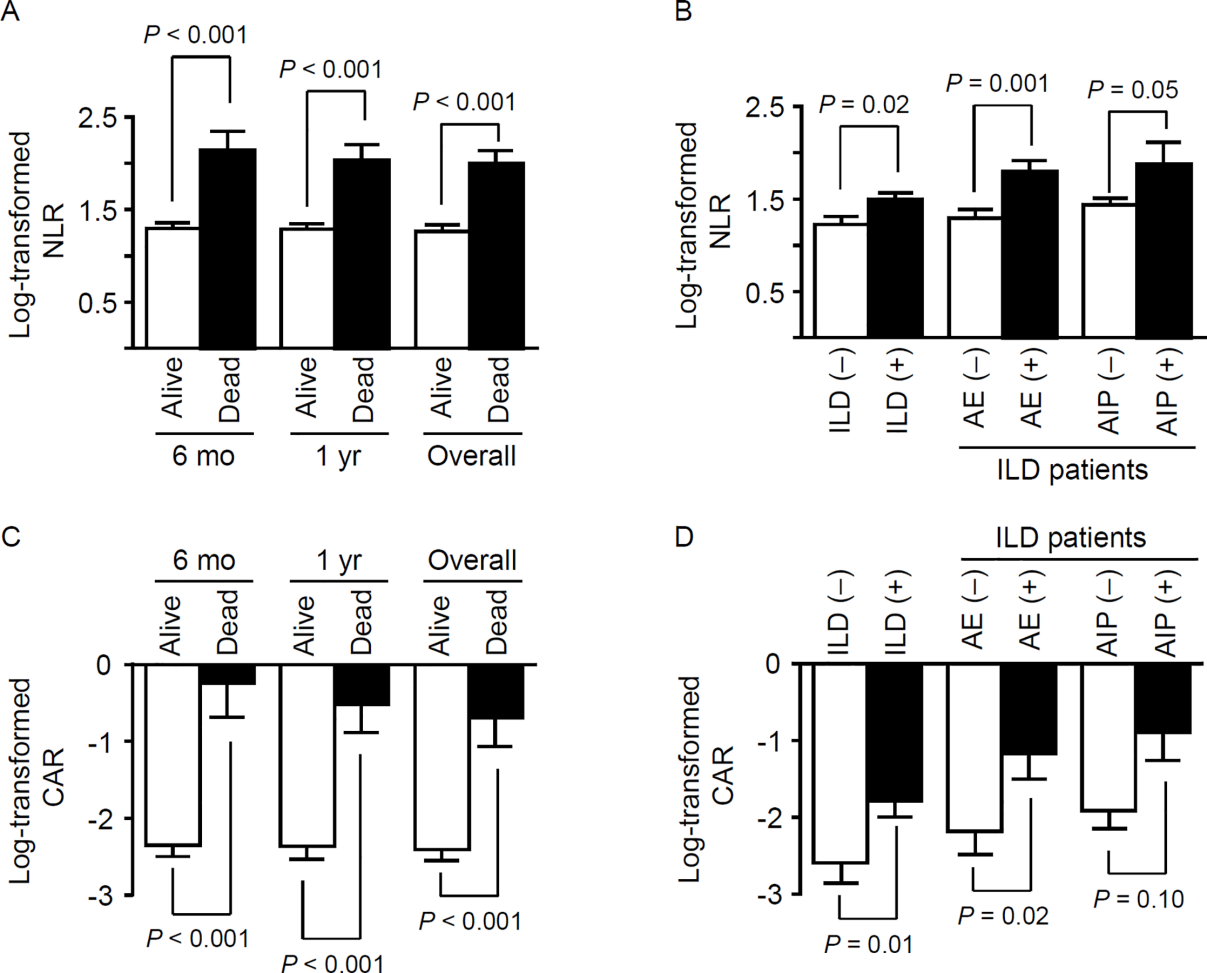
The neutrophil/lymphocyte ratio (NLR) and CRP/albumin ratio (CAR) according to clinically relevant subgroups. The NLR (A and B) and CAR (C and D) were significantly higher in the non-survivor group (versus the survivor group) and in patients with interstitial lung disease (ILD, versus those without ILD). Additionally, among patients with ILD, patients suffering an acute exacerbation (AE) of ILD had significantly higher NLR and CAR values. However, the presence of acute interstitial pneumonitis (AIP) was only associated with the NLR. Bars represent the standard error of the mean, and *P* values were calculated by the t-test.

Among all patients, the NLR had a median of 3.780 (interquartile range [IQR], 2.210–6.275) and the CAR had a median of 0.131 (IQR, 0.029–0.606). The log-transformed NLR (1.266 ± 0.822 versus 2.002 ± 0.684, *P* < 0.001) and CAR (-2.403 ± 2.245 versus -0.687 ± 1.934, *P* < 0.001) values were significantly higher in non-survivors compared to those in survivors. Even when comparing the ratios for survivors and non-survivors at 6 months or 1 year after the IIM diagnosis, the non-survivors had significantly higher log-transformed NLR and CAR values compared to those in the survivors (Fig 2A and 2C).

Although we did not observe an association of the NLR or CAR with the presence of malignancy, the log-transformed NLR (1.225 ± 0.902 versus 1.495 ± 0.761, *P* < 0.05) and CAR (-2.592 ± 2.396 versus -1.781 ± 2.905, *P* < 0.05) values were significantly increased in patients with PM/DM-associated ILD compared to those in patients without ILD (Fig 2B and 2D). Furthermore, among PM/DM patients with ILD, those experiencing AE-ILD showed significantly higher log-transformed NLR and CAR values compared to those in patients who did not have AE-ILD (Fig 2B and 2D). Moreover, the log-transformed NLR was significantly higher in patients with AIP compared to that in patients without AIP.

In bivariate analysis, log-transformed NLR and CAR values were significantly correlated with each other (Pearson correlation coefficient ρ = 0.494, *P* < 0.001). Both ratios were also significantly associated with WBC counts (ρ = 0.496, *P* < 0.001 for NLR; ρ = 0.346, *P* < 0.001 for CAR), hemoglobin levels (ρ = -0.149, *P* = 0.036 for NLR; ρ = -0.295, *P* < 0.001 for CAR), and LDH levels (ρ = 0.229, *P* = 0.002 for NLR; ρ = 0.224, *P* = 0.002 for CAR). Especially, log-transformed CAR values were negatively correlated with FVC percent predicted (ρ = -0.287, *P* = 0.001), DLco percent predicted (ρ = -0.369, *P* < 0.001), ESR (ρ = 0.419, *P* < 0.001), and CPK level (ρ = 0.147, *P* = 0.04).

**Fig 3:**
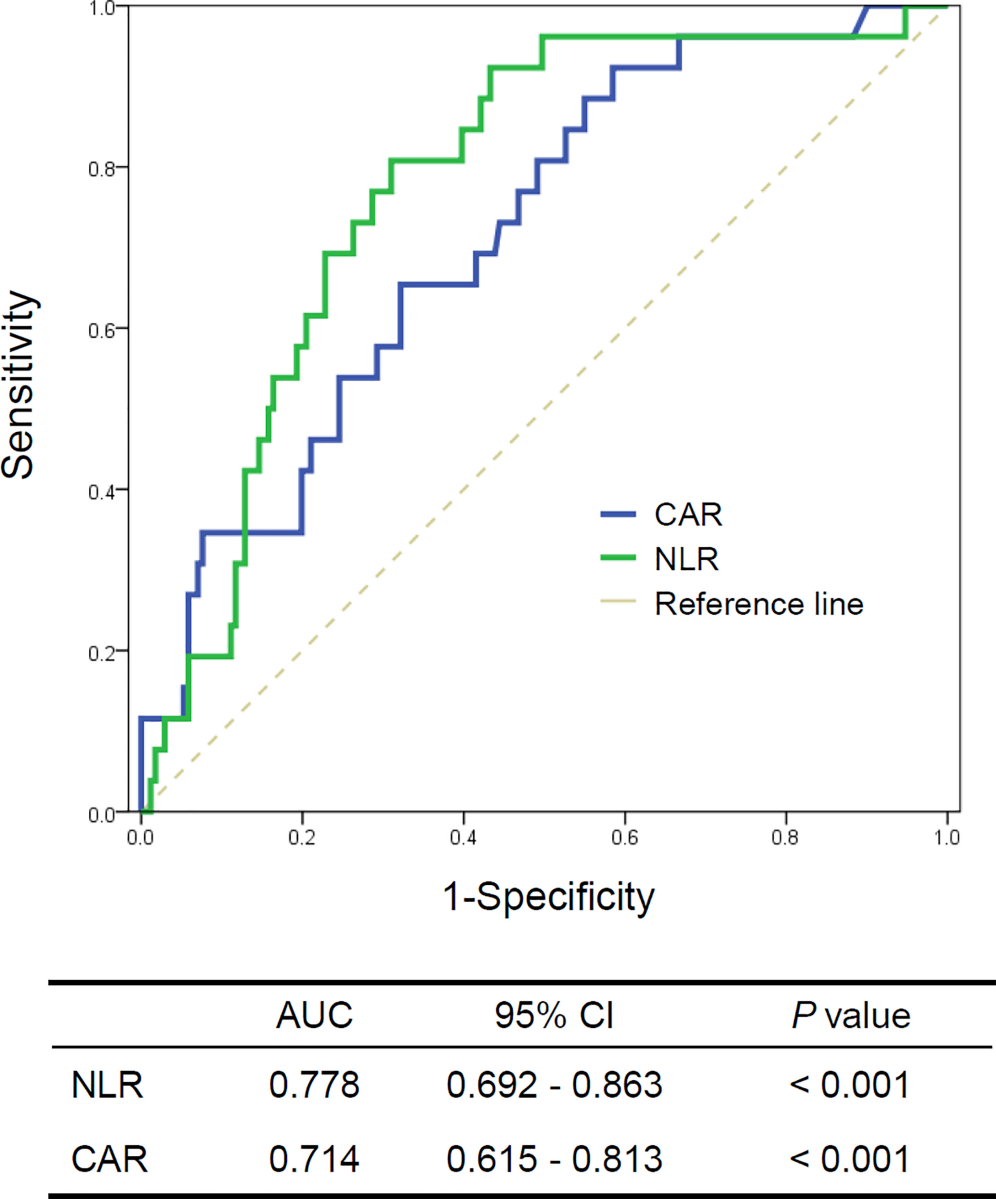
Receiver operator characteristic curves for predicting non-survival in patients with idiopathic inflammatory myopathy. NLR, neutrophil-to-lymphocyte ratio; CRP, C-reactive protein; CAR, CRP-to-albumin ration; AUC, area under the curve; CI, confidence interval.

**Fig 4:**
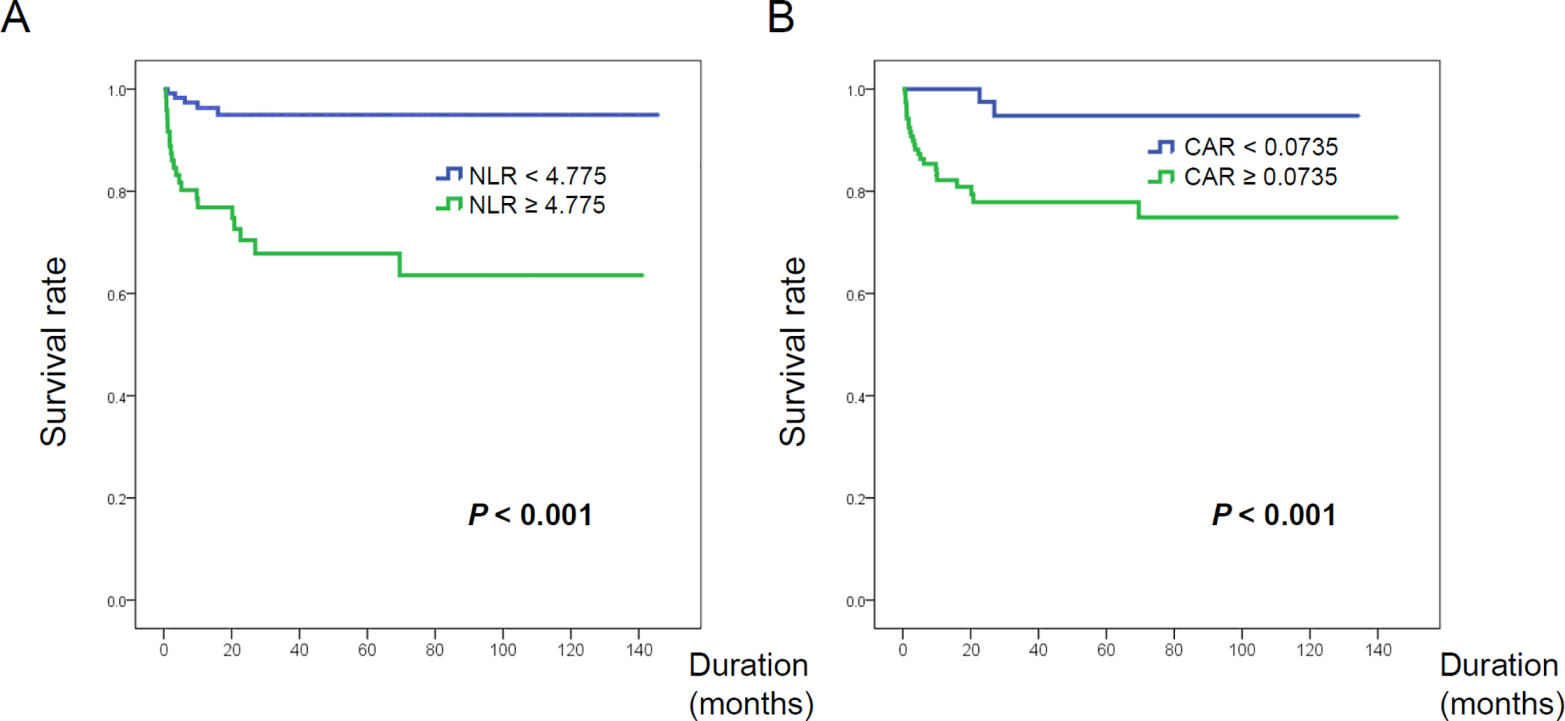
**Survival curves for overall survival in patients with idiopathic inflammatory myopathy, stratified by low/high NLR (A) and CAR (B).** NLR, neutrophil-to-lymphocyte ratio; CRP, C-reactive protein; CAR, CRP-to-albumin ratio.

Since NLR and CAR were significantly associated with non-survival in our patients with PM/DM, their optimal their cutoff levels for predicting mortality were determined by ROC analyses. The mean AUC values of NLR and CAR were 0.778 (95% CI 0.692–0.863) and 0.714 (95% CI 0.615–0.813), respectively (Fig 3). The optimal cut-off values maximizing the sum of the sensitivity and specificity for overall survival were 4.775 for the NLR and 0.0735 for the CAR. When patients with PM/DM were stratified into low and high subgroups according to the optimal NLR and CAR cutoff values, patients with a high NLR or CAR showed significantly worse survival compared to that for patients with low a NLR or CAR (both *P* < 0.001; Fig 4)

Although the proportion of PM/DM patients with ILD was comparable between the high and low NLR subgroups (40.6% versus 59.4%), patients in the high NLR subgroup with ILD were significantly more likely to have AIP (9/41 [22.0%] versus 4/60 [6.7%], *P* = 0.03) and to suffer from AE-ILD (25/41 [61.0%] versus 16/60 [25.0%], *P* < 0.001) compared to patients in the low NLR subgroup with ILD. Among the patients with PM/DM-associated ILD, 15/41 patients (36.6%) in the high NLR subgroup and 5/60 patients (8.3%) in the low NLR subgroup died during the follow-up period (*P* < 0.001).

A high CAR was significantly associated with the presence of ILD (72/124 [71.3%] versus 29/73 [28.7%], *P* < 0.05), AIP (13/72 [18.1%] versus 0/29 [0.0%], *P* < 0.05), and AE-ILD (33/72 [45.8%] versus 7/29 [24.1%], *P* < 0.05). In addition, patients in the high CAR subgroup had a lower FVC percent predicted (70.9% versus 80.2%, *P* < 0.01) and DLco percent predicted (61.8% versus 78%, *P* < 0.001) compared to patients in the low CAR subgroup. However, among the patients with PM/DM associated ILD, the proportion of non-survivors did not significantly differ between the high CAR (18/72 [25.0%]) and low CAR subgroup (2/29 [6.9%]). Finally, the two ratios were not associated with age, the presence of malignancy, or renal function.

### Multivariate analyses of clinical and laboratory characteristics in relation to survival

To determine the independent predictors for survival, we performed a Cox proportional hazard regression analysis with a stepwise forward selection of variables. Malignancies and variables with a *P* value < 0.10 in univariate analyses were entered into the multivariate models (Table 2). In the multivariate analysis, NLR was revealed to be an independent predictor for overall mortality (hazards ratio [HR] = 2.216, 95% confidence interval [CI] = 1.176–3.843, *P* < 0.05 for log transformed NLR; HR = 5.201, 95% CI = 1.923–14.068, *P* = 0.001 for high NLR). Old age (> 50 years), the presence of AIP, and hypoproteinemia (serum protein < 5.5 g/dL) were also significant determinants of overall mortality. However, the presence of malignancy, ANC or neutrophilia (ANC > 7500/mm^3^), ALC or lymphopenia (ALC < 1000/mm^3^), and CAR did not remain as an independent prognostic markers in the multivariate analysis. When multivariate regression analyses were repeated for mortality at 1 year after IIM diagnosis, high NLR was shown to be an independent predictor (HR = 4.541, 95% CI = 1.457–13.601, *P* < 0.01) of mortality within 1 year.

**Table 2:** Cox proportional hazards regression analysis results for overall mortality.

	Univariate Cox regression			Multivariate Cox regression					
				Model 1			Model 2		
	HR	95% CI	*P* value	HR	95% CI	*P* value	HR	95% CI	*P* value
Age	1.073	1.037–1.111	<0.001	1.053	1.012–1.095	0.010	-	-	-
Age > 50 years	6.626	1.988–22.086	0.002	-	-	-	4.507	1.289–15.755	0.018
Fever	2.386	1.036–5.493	0.04	-	-	-	-	-	-
AIP	24.385	11.065–53.742	<0.001	30.119	11.547–78.562	<0.001	14.433	6.076–34.286	<0.001
Cyclophosphamide	2.300	0.867–6.106	0.09	-	-	-	-	-	-
Hemoglobin	0.809	0.647–1.010	0.06	-	-	-	-	-	-
ANC	1.066	1.031–1.103	<0.001	-	-	-	-	-	-
Neutrophilia	2.232	1.012–4.926	0.047	-	-	-	-	-	-
ALC	0.897	0.849–0.948	<0.001	-	-	-	-	-	-
Lymphopenia	3.948	1.759–8.864	0.001	-	-	-	-	-	-
Log-NLR	2.570	1.651–4.002	<0.001	2.216	1.176–3.843	0.01	-	-	-
High NLR	7.640	2.880–20.267	<0.001	-			5.201	1.923–14.068	0.001
CRP	1.125	1.046–1.209	0.001	-	-	-	-	-	-
CRP > 0.5 mg/dL	2.734	1.188–6.291	0.02	-	-	-	-	-	-
Protein	0.388	0.246–0.613	<0.001	0.441	0.232–0.838	0.01	-	-	-
Protein < 5.5 g/dL	3.787	1.518–9.448	0.004	-			3.586	1.333–9.650	0.01
Albumin	0.329	0.192–0.564	<0.001	-	-	-	-	-	-
Albumin < 3.5 g/dL	3.071	1.290–7.313	0.011	-	-	-	-	-	-
Log-CAR	1.437	1.173–1.760	<0.001	-	-	-	-	-	-
High CAR	8.199	1.936–34.716	0.004	-	-	-	-	-	-
Creatinine	3.758	1.543–9.154	0.004	4.375	1.840–10.401	0.001	-	-	-
ILD	3.561	1.428–8.880	0.006	-	-	-	-	-	-
AE-ILD	7.930	3.585–17.541	<0.001	-	-	-	-	-	-
Malignancy	1.089	0.410–2.888	0.86	-	-	-	-	-	-

All continuous variables were maintained in model 1. In model 2, continuous variables were converted to dichotomous variables using clinically relevant or the ROC-derived cut-points. Neutrophilia was defined as an ANC > 7500/mm^3^ and lymphopenia as an ALC < 1000/mm^3^. NLR or CAR values were dichotomized into high and low groups using a cut-off point of 4.775 and 0.0735, respectively.

Abbreviation: HR, hazard ratio; CI, confidence interval; AIP, acute interstitial pneumonia; ANC, absolute neutrophil count; ALC, absolute lymphocyte count; NLR, neutrophil-to-lymphocyte ratio; CRP, C-reactive protein; CAR, CRP-to-albumin ration; ILD, interstitial lung disease; AE-ILD, acute exacerbation of ILD.

## Discussion

In the present study, patients with PM/DM in the non-survivor group exhibited a significantly higher baseline NLR compared to that in the survivor group. Moreover, high NLR was an independent and significant risk factor for overall survival in patients with PM/DM. Especially, a high NLR was associated with lung involvement, which is associated with a high mortality rate; approximately a third of patients with PM/DM-associated ILD and a high NLR suffered from AE-ILD in the present study.

Based on the recent studies, the 5-year survival rates of patients with PM/DM are estimated to be 70–96% [5]. This is similar to the 5-year survival rate (82%) obtained in the present study. These figures are notably higher compared to those reported 30–40 years ago, as the earlier diagnosis and use of immunosuppressive agents has progressively improved the outcomes [2,5]. Nevertheless, the common causes of death in PM/DM appear to have not changed over time. Cancer, pulmonary complications, cardiovascular diseases, and infections are generally cited as the main causes of mortality [25]. Similarly, in the current study, infection, pulmonary complications (including AE-ILD), and cardiovascular events were the most frequent primary causes of death (S1 Table).

The frequency of ILD in patients with PM/DM has been widely reported (8.6–85.6%), and a meta-analysis of 23 studies revealed that 834/2079 patients with PM/DM (40.1%) had ILD [26]. ILD, as a frequent extra-muscular manifestation, leads to increased morbidity and mortality in patients with PM/DM [7]. Especially, patients with an acute type of ILD have dismal outcomes because of resistance to conventional therapy; the mortality rate is reported to be 50–100% [27,28]. Additionally, an acute exacerbation of pre-existing ILD was one of the most common causes of death, as well as pneumonia, in an autopsy study [29], and is the main reason requiring intensive care in patients with PM/DM [30]. In the present study, the non-survivor subgroup had significantly more patients with an acute-type or acute exacerbation of ILD.

Because patients with IIM, especially those with PM/DM-associated ILD, follow variable clinical courses, it is a great clinical challenge to predict their outcome at the diagnosis. Acute/subacute ILD, older age, and a lower level of FVC were shown to be associated with poor survival in patients with PM/DM-associated ILD [31]. Myositis-specific autoantibodies also provide prognostic information, beyond several clinical predictors of PM/DM-associated ILD. Anti-Jo-1, anti–PL-7 and anti–PL-12 antibodies could increase the risk of ILD. Furthermore, Anti-CADM-140/MDA-5 antibody is reported to be positively associated with the presence of rapidly progressive ILD while anti-155/140 targeting TIF-1 is negatively associated with the presence of ILD [32]. Concerning non-antibody biomarkers related with prognosis, serum ferritin, KL-6, and chemokine CXCL9/10 levels may be useful markers for ILD activity or severity [33]. However, these biomarkers, except for anti-Jo-1 antibody and ferritin, are not commonly available at routine clinical practices. Therefore, an unmet clinical need clearly exists for easily accessible biomarkers with prognostic value in PM/DM.

The NLR is easily obtained by simply dividing the number (or proportion) of neutrophils by that of lymphocytes, and is calculated from the differential white cell count test routinely performed in most clinical settings. The NLR has been reported to have prognostic value or to be associated with disease activity in many malignant and non-malignant diseases, including several systemic autoimmune diseases [13–15,34–37]. Recently, two studies in China revealed that the NLR was associated with the physician-reported IIM disease activity and the presence of DM-associated ILD [17,18]. In the present study, a significant association was observed between a high NLR and the presence of ILD or an episode of acute exacerbation, consistent with the results of the study by Yang et al [18]. Interestingly, the NLR has also been reported to be significantly related to exacerbation in patients with chronic obstructive pulmonary disease [38]. The relationship between the NLR and lung involvement could contribute to its predictive value for poor outcomes in patients with PM/DM. To the best of our knowledge, the present study is the first to show that NLR can predict overall survival in patients with PM/DM.

Although the NLR is associated with a worse prognosis in various diseases, little is known regarding the underlying biology or mechanisms. Woo et al. reported that the NLR is negatively associated with phytohemagglutinin-induced levels of IFN-γ, but is not associated with nil response [39]. In addition, the NLR is positively correlated with the expression of proinflammatory IL-17 [40]. CD4+interferon (IFN)-γ+ cells and the intracellular IFN-γ/interleukin (IL)-4 ratio in CD4+ cells are significantly lower in patients with DM [41]. Moreover, the disruption of IFN-γ in C protein-induced myositis (CIM), a mouse model of PM, leads to up-regulation of IL-17A, exacerbating the disease with neutrophil infiltration into the sites of inflammation [42]. Furthermore, IL-17 expression is increased in the muscle and blood of patients with DM [43,44]. These findings suggest that NLR could reflect a skewed cytokine microenvironment associated with poor prognosis in patient with PM/DM.

Patients with active DM have a lower number of circulating T cells [45], and lymphopenia is reportedly associated with a poor outcome and the presence of ILD in patients with PM/DM [30,46]. In the present study, lymphopenia was significantly more prevalent in the non-survivor group than in the survivor group, and was associated with overall mortality in a univariate analysis. However, lymphopenia did not remain significant in our multivariate models. Recent evidence shows that neutrophils are actively involved in both innate and adaptive immunity, and play an important pathogenetic role in many autoimmune diseases. Although the role of neutrophils in IIM has not been deeply investigated, patients with PM/DM have an increased number of low density granulocytes (LDCs), a proinflammatory subtype of human neutrophils, in peripheral blood [47]. In addition, patients with PM/DM-associated ILD have significantly an enhanced formation of neutrophil extracellular traps, and the decreased degradation of neutrophil extracellular traps can directly prime T cells [48]. Interestingly, Kalra et al. recently reported that the NLR is correlated with the frequency of circulating LDGs and is associated with liver-related death in patients with liver cirrhosis [49]. Thus, the NLR may be a simple index reflecting qualitative and quantitative change of both neutrophils and lymphocytes.

As described in S1 Table, most deaths of our study subjects were attributed to ILD aggravation or malignancy (13/26, 50%). Anti-MDA5 and anti-TIF1-γ antibodies are related with rapidly progressive ILD or malignancy-associated IIM, respectively [32,50]. Therefore, our patients with early death would be more likely to have these antibodies and high NLR values could be associated with a distinct serological subset of IIM. However, unfortunately, myositis-specific autoantibodies were not investigated in the present study.

The NLR reference range has not been extensively studied. Kweon et al. reported that the median NLR values are 1.53 and 1.54 for Korean men and women, respectively, and the upper 2.5^th^ percentile values are 3.51 and 3.54, respectively [51]. In Belgian subjects, the upper limit of the 95% CI is 3.53, with a mean of 1.65 [52]. Therefore, patients with IIM have an NLR beyond the reference range in the present study (median, 3.78) and in the study by Yang et al. (mean, 4.50) [18]. However, Gao et al. reported a much lower NLR in patients with IIM (median, 2.88) [17]. Moreover, the optimal NLR cut-off level for predicting mortality has been variously reported in diverse conditions. In most studies of patients with non-infectious and non-malignant diseases, the NLR cut-off value associated with mortality has been reported to be about 5.0 [53–56]. Similarly, the optimal NLR value associated with overall mortality in patients with PM/DM was 4.775 in the present study. However, additional studies are needed to further establish the optimal NLR cut-off value for predicting poor outcomes in patients with IIM.

The present study has several limitations to consider. First, the study was retrospective in nature and the number of non-survivors was relatively small, which may limit the actual prognostic value of the NLR. Large-scale prospective multi-center studies are warranted to validate the present results. Second, the follow-up duration was not long enough to assess the late mortality. Survivors had a mean of 3.1 years of follow-up and 22 survivors had anti-Jo-1 positive ILD. Patients with anti-Jo-1 or anti-aminoacyl-tRNA synthetase (ARS) positive ILD tend to be stabilized with immunotherapy but their pulmonary function could be slowly deteriorated over 5 to 10 years, leading to death [57,58]. In the current study, because most deaths occurred within 1 year after the diagnosis (20/26, 76.9%), the predictive potential of baseline NLR may be confined to patients with early mortality. Third, myositis-specific autoantibodies, known to have prognostic impact, were not evaluated in all patients, with the exception of anti-Jo-1 antibodies. The simultaneous evaluation of pro-inflammatory cytokines and myositis-specific autoantibodies could provide additional insight into the clinical implications of the NLR or underlying mechanism to explain its association of poor outcome in patients with PM/DM.

In conclusion, the present study demonstrated that the baseline NLR is an independent and significant indicator of overall death in patients with PM/DM, and a high NLR is associated with the presence of ILD and AE-ILD. As a simple and inexpensive index, the NLR could be a useful marker for predicting the prognosis of patients with PM/DM, along with previously identified risk factors, when making treatment-related decisions.

## Supporting information

S1 Table Summary of causes of death in our population (n = 26). (DOCX)
