# Supplementary material for: Baseline peripheral blood neutrophil-to-lymphocyte ratio could predict survival in patients with adult polymyositis and dermatomyositis: A retrospective observational study
Source: PLoS One. 2018 Jan 2;13(1):e0190411. doi: 10.1371/journal.pone.0190411 (PMC5749807; doi:10.1371/journal.pone.0190411)
Supplement: S1 Table — (DOCX) [file pone.0190411.s001.docx]

**Supporting information**

**S1 Table**. Summary of causes of death in our population (n = 26)

| Cause | n |
| --- | --- |
| ILD aggravation with infection | 6 |
| ILD aggravation | 4 |
| Pneumonia | 3 |
| Pulmonary complication after lung transplantation | 3 |
| Sudden cardiac arrest | 2 |
| Respiratory arrest with malignant pleural effusion | 2 |
| Septic shock | 2 |
| Connective tissue disease | 1 |
| Infective endocarditis | 1 |
| Lung cancer | 1 |
| Respiratory failure due to diffuse alveolar hemorrhage | 1 |
